# Epidemiology of accelerometer-based sleep parameters in US school-aged children and adults: NHANES 2011–2014

**DOI:** 10.1038/s41598-022-11848-8

**Published:** 2022-05-10

**Authors:** Shaoyong Su, Xinyue Li, Yanyan Xu, William V. McCall, Xiaoling Wang

**Affiliations:** 1 Georgia Prevention Institute, Medical College of Georgia, Augusta University, Building HS-1715, Augusta, GA 30912 USA; 2 School of Data Science, City University of Hong Kong, Hong Kong, China; 3 Department of Psychiatry and Health Behavior, Medical College of Georgia, Augusta University, Augusta, GA USA

**Keywords:** Epidemiology, Risk factors

## Abstract

We aimed to provide objectively measured sleep parameters across lifespan by sex and race in a national representative sample of US population. The study included 11,279 participants 6 years and older from the National Health and Nutrition Examination Survey (NHANES) 2011–2014, who had at least 3 days of valid sleep parameters calculated from 7-day 24-h accelerometer recording. Sleep duration showed a U-shaped association with age and reached the minimum at age 40 and started to increase again around age 50. The clock time for sleep onset (CTSO) delayed with age and reached the maximum at about age 20. CTSO then advanced until age 50, leveled off until age 70, then advanced again after age 70. Sleep efficiency showed an overall decreasing trend across the lifespan but stabilized from age 30 to about age 60. US young adults in age 20 s are the ones who slept at the latest around midnight, while the middle aged US residents between 40 and 50 years old slept the least. Females generally present longer sleep duration than males, while more likely to have later sleep onset, particularly at older ages. Non-Hispanic Blacks showed worse sleep characteristics, i.e. sleep later, sleep shorter, and sleep less efficiently, compared to other racial groups. In conclusion, this study provides valuable insights on the characteristics of sleep habits of residents of the United States by using objectively measurements of sleep parameters and will help guide personalized advice on sleep hygiene.

## Introduction

Sleep is a critical component of human biologic needs and essential functions^1^. Good sleep helps to promote optimal health and quality of life. Changes in sleep quality, quantity, and timing strongly influence physical and mental health, as well as daily performance. For example, habitual short sleep duration (< 7 h) has been associated with increased risk of multiple adverse health conditions including obesity, diabetes, hypertension, cardiovascular disease, mental disorders and cognitive dysfunction, as well as all-cause mortality^2^. On the other hand, long sleep duration (> 9 h) may also be harmful in certain populations (e.g. older adults > 64 years old)^3^. Lower sleep efficiency and later sleep timing have also been associated with multiple adverse health outcomes^4–6^.

Sleep parameters change across the human life span. A nationally representative sample is required to unbiasedly estimate these age-related changes as well as the potential modifier effects of demographic factors such as sex and race. Previous national survey data (or large aggregated data over multiple samples from the general population)^7–9^ on sleep duration and sleep quantity have been collected using self-report questionnaires. The findings are relatively consistent with sleep duration decreasing from birth to 45–55 years old and sleep efficiency decreasing continuously with age across the life span. Limited information is available for sleep onset, i.e. timing of sleep, because this parameter is difficult to be accurately captured from self-report sleep questionnaires. In recent years, research-grade activity monitors (i.e. accelerometers) have been widely used in large-scale population studies to provide more objective and cost-effective estimates of sleep parameters derived from rest-activity data. However, there is few research dedicated to objective measures of sleep parameters in nationally representative samples. Accelerometer was included in the National Health and Nutrition Examination Survey (NHANES) 2003–2006. However, participants were only asked to wear the device during the daytime. Based on the nighttime non-wear period, Urbanek et al.^10^ estimated the age- and gender-distribution of bedtime and chronotype in US adolescents and adults. In the current study, we propose to objectively estimate the Clock time for sleep onset (CTSO), sleep duration and sleep efficiency by using data from the NHANES 2011–2012 and 2013–2014 cycles, which collected 7-day 24-h accelerometer data in 11,279 participants (aged 6 and older). The goal is to provide US nationally representative objective estimates of sleep parameters by age, sex and race.

## Method

### Study population

**Figure 1:**
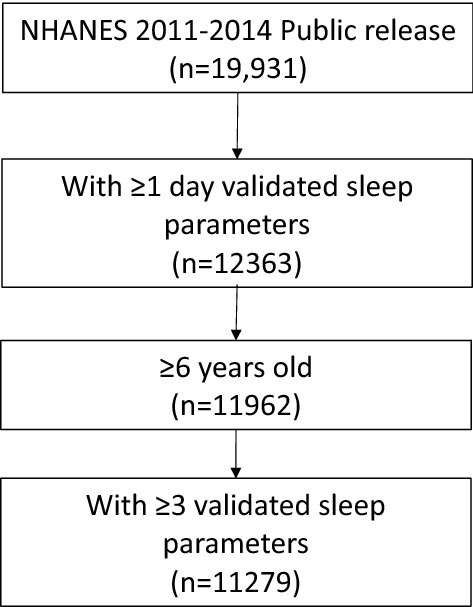
Flowchart for inclusion of study participants.

This study was conducted using data from the NHANES survey 2011–2012 and 2013–2014 cycles. These two cycles were selected due to the availability of 24 h accelerometer data. NHANES used a multistage probability sampling design to produce a weighted, representative sample of the US population^11^. The National Center for Health Statistics Research Ethics Review Board approved all NHANES protocols, and all participants gave informed consent. All methods were performed in accordance with the relevant guidelines and regulations. Our sample included participants with age ≥ 6 years old that had at least 3 days of validated sleep parameters calculated from accelerometer recording (*n* = 11,279). Figure 1 illustrates the flow of participants selected for inclusion in this analysis.

### Accelerometer recording and data preprocessing

All participants aged 6 years and older during the 2011–2012 cycle and all participants aged 3 years and older during the 2013–2014 cycle were asked to wear an accelerometer (ActiGraph Model GT3X + , ActiGraph of Pensacola, FL) all day and night for 7 consecutive days. The device was worn on the non-dominant wrist, if possible. Raw signals obtained on the x-, y-, or z-axes every 1/80 of a second (80 Hz) were processed, flagged and summarized at the minute level and released by NHANES in November 2020. These summary measures in the minute summary file (PAXMIN) are specified in Monitor-Independent Movement Summary (MIMS) units, which is a non-proprietary, open-source, device-independent universal summary metric developed by researchers at Northeastern University^12^. MIMS triaxial value (variable name: PAXMTSM) at the every minute level was used to calculate sleep parameters. MIMS triaxial values were changed to missing (i.e. a value of 0) if they met any of the following conditions: (1) PAXMTSM is coded as "-0.01"; (2) estimated wake/sleep/wear status during the minute (variable name PAXPREDM) is coded as “Non wear”; or (3) minute data quality flag count (variable name PAXQFM) is larger than “0”. R package “accelmissing”^13^ was used to impute the missing count values in the accelerometer data with the following pre-processing steps: (1) the minimum minutes of missing interval were defined as 60 min; (2) the valid days were defined as more than 16 h of wearing; and (3) the minimum number of valid days that the subject should have was defined as 4 days.

### Sleep parameters

Sleep parameters were derived from accelerometer data using an unsupervised sleep–wake identification algorithm based on Hidden Markov Model (HMM) to infer the sequence of “hidden states” of sleep or wake for each individual^14^. The block of the longest sleep period in the day (noon-noon) was identified as the sleep period time (SPT) window. The start of SPT window was defined as the sleep onset time. To enable the correlations of values from a 24 h clock in a linear space, a simple transformation was done for the sleep onset time after 12 am, such that 1am past midnight was considered as 25 h. Wake/activity bouts were identified during the SPT window. Sleep duration was defined using the following equation: sleep duration = the SPT window duration –the summed duration of all wake bouts. Sleep efficiency was calculated as sleep duration divided by the SPT window duration. This HMM can be directly applied to summary activity count data, which is the case for the NHANES released data. R code for implementing the HMM algorithm is at https://github.com/xinyue-L/hmmacc. Records with a SPT window duration < 3 h or > 15 h were excluded before the calculation of average sleep parameters for each individual. Individuals with valid sleep parameters less than 3 days were excluded from the analysis.

### Age, sex and race

Age, sex and race information were obtained from the demographic file. Race was classified into 4 groups: Non-Hispanic (NH) White, NH Black, Mexican American and other race (i.e. other Hispanic, Asian and other ethnicity).

### Statistical analysis

To account for complex survey design and produce representative estimates of the US population, analyses were conducted using the survey data analysis in STATA (v16). Four-year survey weights were calculated and used in all analyses to adjust for unequal selection probability and non-response bias in accordance with NHANES analytical guidelines^15^. Population means, proportions, and standard errors (SE) were estimated and reported. Survey weighted linear regression was used to assess the distribution of sleep parameters across age, sex and race. Due to the nonlinear relationship between age and sleep parameters, linear, quadratic, cubic and quartic trends were fitted by including age, age^2^, age^3^ and age^4^ in the regression model to understand the changes of sleep parameters with age. The interactions between sex and age (i.e. linear, quadratic, cubic and quartic) or between race and age were also tested to examine whether the associations of sleep parameters with age were modified by sex or race. Because the distribution of sleep efficiency is negatively (or left) skewed, sleep efficiency was further divided into quartiles and survey weighted ordinal logistic regression was conducted to confirm the findings observed from the linear model. Statistical significance was set at *p* < 0.05.

## Results

Our analytical samples include 11,279 participants aged >  = 6 years (mean ± SE: 41.6 ± 0.48 years), representing 200.3 million noninstitutionalized residents of the United States. Distributions of age, sex and race as well as sleep parameters are presented in Table 1.

**Table 1:** General characteristics of the participants (*n* = 11,279).

Variables	Values
Age, years	41.6 ± 0.48
Female, %	53.08
**Race, %**	
NH white	64.44
NH black	11.48
Mex American	10.1
Other race	13.97
Sleep duration, hours	8.19 ± 0.02
Clock time of sleep onset	22.84 ± 0.03
Sleep efficiency	0.96 ± 0.001

For continuous traits, data are present as mean ± SE.

*NH* Non-Hispanic, *Mex* Mexican.

The distributions of CTSO, sleep duration and sleep efficiency by age groups are presented in Table 2. The median CTSO ranged from 10:14 pm to 11:49 pm for different age groups. Furthermore, 25% of children aged 6–13 had a CTSO close to 11:00 pm (i.e. 10:58 pm) and this time increased to 12:00 am (i.e. 12:06 am) and 1:00 am (i.e. 0:58 am) for children aged 14–17 and young adult aged 18–25, respectively. The median sleep duration ranged from 7 h 50 m to 8 h 58 m and the median sleep efficiency ranged from 0.956 to 0.977 for different age groups.

**Table 2:** Distribution of clock time of sleep onset, sleep duration and sleep efficiency by age groups.

Age Group	N	Clock time of sleep onset (CTSO)						Sleep duration, hours						Sleep efficiency					
		5%	10%	25%	50%	75%	90%	5%	10%	25%	50%	75%	90%	5%	10%	25%	50%	75%	90%
6–13	2509	20.82	21.14	21.67	22.24	22.97	23.70	7.69	8.01	8.46	8.97	9.48	9.86	0.95	0.96	0.97	0.98	0.98	0.99
14–17	847	21.41	21.77	22.37	23.15	24.01	24.94	6.90	7.24	7.77	8.29	8.93	9.61	0.93	0.94	0.96	0.97	0.98	0.99
18–25	1047	21.53	22.03	22.88	23.82	24.96	26.00	6.53	6.98	7.50	8.22	8.97	9.51	0.88	0.91	0.94	0.96	0.98	0.98
26–35	1148	20.93	21.48	22.40	23.25	24.13	25.30	6.43	6.78	7.37	8.01	8.78	9.42	0.89	0.92	0.95	0.97	0.98	0.98
36–45	1277	20.53	21.28	22.07	22.84	23.68	24.53	6.27	6.65	7.28	7.90	8.54	9.32	0.88	0.91	0.95	0.97	0.98	0.98
46–55	1313	20.20	21.05	21.91	22.71	23.58	24.62	6.21	6.54	7.18	7.83	8.62	9.41	0.87	0.91	0.95	0.96	0.98	0.98
56–65	1385	20.13	20.65	21.70	22.62	23.58	24.35	5.98	6.52	7.19	7.91	8.71	9.51	0.89	0.91	0.94	0.96	0.98	0.98
66–75	1011	19.86	20.53	21.51	22.46	23.38	24.33	6.38	6.79	7.44	8.24	9.14	9.94	0.87	0.90	0.94	0.96	0.97	0.98
> = 76	742	20.04	20.60	21.48	22.40	23.19	23.99	6.46	6.80	7.53	8.26	9.10	9.85	0.87	0.90	0.93	0.96	0.97	0.98

**Figure 2:**
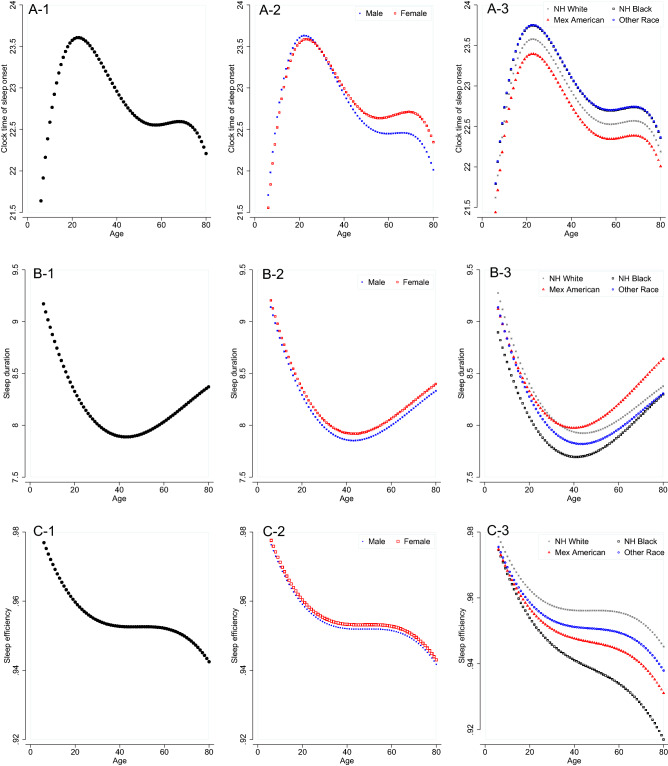
Age, sex and race distribution of clock time of sleep onset (CTSO), sleep duration and sleep efficiency. (**A**) 1–3 for CTSO. Please note that the curve for Other Race is overlapped with the curve of NH Blacks; (**B**) 1–3 for sleep duration and (**C**) 1–3 for sleep efficiency.

**Figure 3:**
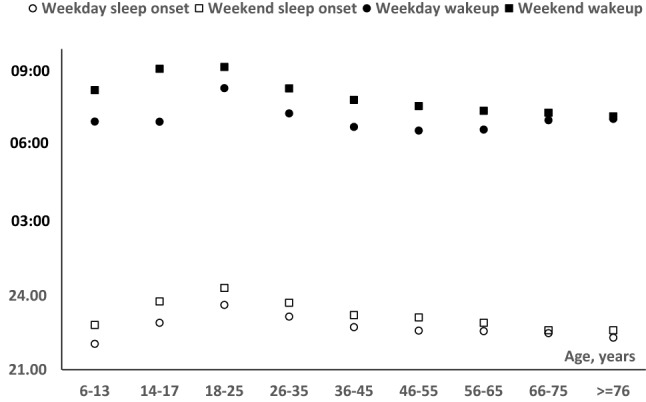
Weekday vs. Weekend sleep onset and wakeup time across the life span.

Figure 2 displays the changes in sleep parameters with age and the potential modifying effect of sex and race. As expected, CTSO delayed with age and reached the maximum at about age 20. CTSO then advanced until age 50, leveled off until age 70, then started to advance again after age 70 (Fig. 2A-1). In addition to a main effect of sex (beta = 0.076, p = 0.042, which suggests that females had a 5 min later CTSO than males controlling for all other factors), there was also a significant interaction between sex and age. As shown in Fig. 2A-2, the delay in CTSO with age was faster in males than in females before age 20. There is also a significant main effect of race (*p* = 0.0003) with Mexican Americans having the earliest CTSO (~ 11 min earlier than NH Whites) and NH Blacks and other race having the latest CTSO (~ 10 min later than NH Whites). The differences between Mexican Americans and NH blacks or other race reached statistical significance (*Ps* < 0.001). A significant interaction between race and age was also identified. As shown in Fig. 2A-3, this interaction was mainly driven by a slower increase in Mexican Americans and a faster increase in NH Blacks and other race in the CTSO with age than the NH Whites before age 20.

Also as expected (Fig. 2B-1), sleep duration decreased with age and reached the minimum at about age 40 and started to increase again around age 50. There was a main effect of sex (*p* = 0.004) with females on average having 4 more minutes of sleep than males controlling for all other factors. There was also a main effect of race (*p* < 0.0001) with NH blacks on average having 15 min less sleep than NH Whites and Mexican Americans (*ps* < 0.001) and 8 min less sleep than other race (*p* = 0.012). A significant interaction between race and age was also identified. As shown in Fig. 2B-3, this interaction was mainly driven by a faster decrease in the sleep duration with age in NH Whites than in the Mexican Americans and NH Blacks before age 40.

As shown in Fig. 2C-1, sleep efficiency showed a sharp decrease with age until age 30, then it leveled off until age 60, with another sharp decrease after age 60. No significant main effect of sex or interaction between sex and age was identified. A significant main effect of race (*p* < 0.0001) was identified with NH Whites having a higher sleep efficiency than NH Blacks (*p* < 0.001), Mexican Americans (*p* < 0.001) and other race (*p* = 0.001) and NH Blacks having a lower sleep efficiency than Mexican Americans (*p* = 0.002) and other race (*p* < 0.001). A significant interaction between race and age was also identified. As shown in Fig. 2C-3, this interaction was mainly driven by a faster decrease in the sleep efficiency with age in NH Blacks and Mexican Americans than NH Whites and other race before age 30.

The weekend vs. weekday sleep onset time and wakeup time across different age groups are displayed in Fig. 3. Later sleep onset and later wakeup during the weekend in comparison with the weekday only happen for age groups that go to school or work. The median weekday-weekend difference for sleep onset time was 46 min for children aged 6–13, peaked in children aged 14–17 (median difference of 52 min) and was around 30 min for college students and working adults. The median weekday-weekend difference for wakeup time was 77 min for children aged 6–13, also peaked in children aged 14–17 (median difference of 129 min) and was around 60 min for college students and working adults.

## Discussion

In the present study, we provided the first report of the objective estimates of sleep parameters based on actigraphy data collected from 7 consecutive days in a nationally representative sample of the US. As expected, sleep varies across age, sex, and racial groups. American young adults in age 20 s have the latest CTSO (around midnight), while the middle aged Americans between 40 and 50 years old sleep the least. Our finding that CTSO advances from age 20 through late in life is consistent with what we have previously reported in an actigraphy study of depressed adults, with CTSO advancing approximately 4 min per year^16^. Sleep efficiency shows an overall decreasing trend across the lifespan but stabilized from age 30 s to about age 60 s. Females generally present longer sleep duration than males, while more likely to have later sleep onset, particularly at older ages. In this report, NH Black Americans show worse sleep characteristics, i.e. later CTSO, sleep shorter, and sleep less efficiently, compared to other racial groups.

It is acknowledged that, in general, sleep duration at night decreases with age. However, this trend is more evident from childhood to adulthood, but less significant within the older population (age > 60)^17,18^. A recent meta-analysis in actigraphy-assessed sleep characteristics revealed that sleep duration was shorter at older ages only until approximately age 50, and was slightly longer at ages above 50 years^19^. However, given the exploratory nature of the study, there was no solid conclusion. In current study, based on actigraphy data, we observed a U-shaped curve of sleep duration across the lifespan, with sleep duration decreasing significantly from age 10 s to age 50 s, before slightly increasing again in the older age groups. Our finding is supported by a recent large population study consisted of 68,640 Japanese residents ranging from adolescents to the elderly (10–89 years old), in which sleep duration exhibited a U-shaped pattern with increasing age, with nadir of the curve in middle age^20^. Similar pattern was also found in a nationally representative French sample of 24,671 subjects aged 15–85 years old^9^. This rising tendency of sleep duration in the elderly may be due to the fact that most people retire between 60 and 65 years of age, and thus no need to get up as early as those in middle age who are still working. However, it should be of note that older adults with chronic diseases tend to report more fatigue and lethargy, which may lead to long sleep^21^. Therefore, prolonged sleep duration in the elderly may also be associated with poor health. It warrants prospective and longitudinal studies to understand the pattern of sleep duration in the elderly and its association with health.

In addition to how much people sleep, when people sleep (i.e. the time of sleep onset) is also important. In this study, we found that sleep onset is significantly delayed during the transition period from childhood to adolescence and young adulthood, reaching the “lateness” peak at around the age of 20, then gradually advanced with age. Our findings are generally consistent with previous studies conducted in different countries, suggesting a minimum difference across cultures^19,22^. Moreover, our results revealed that in the US children at school age, particularly high school students, exhibited the largest weekday-weekend differences for sleep onset and wakeup time. For example, a 16 years old of high school student may fall asleep at 11:45 pm and wake up at 9am in school-free days. However, in school days, the student may sleep a little bit earlier but still late at 11:00 pm, but has to wake up at 7am in the morning, showing a huge gap of 1 h 15 min in sleep time. Recent studies have suggested that the delayed timing of sleep/wake in adolescents may be due to the increased social demands and behavioral changes at this age, especially the inappropriate use of electronic media before sleep^23^. However, it cannot be ruled out of the biological changes in circadian system and sleep/wake homeostasis that both occur during adolescence^24,25^. This mismatch between sleep–wake behavior and associated physiological rhythms may lead to a cumulative sleep debt with fatigue, behavioral problems and poor academic achievement^26^. Further studies are needed to characterize the epidemiology of the objective estimate of this sleep variation in the US adolescents and examine the potential mechanisms.

The third sleep characteristic presented in this study is sleep efficiency, a vital measure of sleep quality. There is an overall agreement that sleep efficiency decreases with age, although some different findings exist between our study and previous studies. Recent meta-analysis and population-based studies have suggested a linear decline of sleep efficiency with increasing age^19,20,27^. In our study, on the contrary, sleep efficiency showed a sharp decrement with age before the age of 30 and after the age of 60, but remaining relatively stable across the middle-aged adulthood (ages 30–60). This inconsistency may be due to difference in study design, populations, and measurement modality. For example, Sleep Heart Health Study enrolled 5407 men and women aged 40–99 from multiple study centers, and assessed sleep characteristics by using the objective unattended home polysomnography (PSG) and subjective sleep questionnaires^27^, while the current study is of utilizing wrist actigraphy-based data for sleep estimates in a nationally representative sample, which allows for a greater number of nights studied and increased environmental validity of home-based assessment. Furthermore, in most previous studies, sleep efficiency was simply defined as the ratio of total sleep time to total time in bed^28^. In the present study, we first identified the sleep period time (SPT) window within each 24 h according to actigraphy data, then identified accountable wake/activity bouts during this window. Sleep efficiency was calculated as (the SPT window duration – the summed duration of all wake bouts) divided by the SPT window duration. Our data suggest that American adults may maintain sleep efficiency at a certain level for a long period through adulthood, although they may have the least sleep at middle age.

In addition, we found sex differences in sleep duration and CTSO, but not for sleep efficiency. In line with previous reports and the most recent study using the questionnaire data from NHANES 2017-March 2020 Pre-Pandemic cycle^29^, females tend to sleep on average longer than males, especially from adulthood onwards. Burgard and Ailshire analyzed a sleep-diary data from nationally representative samples of working-age adults in the American Time Use Surveys of 2003–2007^7^. Overall and at most life course stages, women slept more than men. However, this difference varied with work and family responsibilities. Women were more likely to experience sleep interruption for caregiving work, particularly among parents of young children. The overall female advantage in sleep time is tempered by greater burden of interrupted sleep that women face and the larger male advantage in leisure time that exists throughout midlife. Similar to previous studies^20,30^, our data also suggested women over age 50 years slept later than men at the same age, with an overall mean difference of 15.3 min in sleep onset after age 50 years. This difference may be due to the changes in women’s role in the home and workplace^31^ and the biological changes during peri- and post-menopausal period^32^, which needs further studied. An unexpected finding is that we didn’t observe difference in sleep efficiency between males and females. This is in contrast to previous studies in which women, especially in older age, are more likely to report worse sleep quality, less sleep efficiency and/or more sleep disturbance. It is of note, however, that objective assessment of sleep may not predict subjective measures of sleep quality^33,34^. Further studies are needed to discover the biological correlates of subjective sleep quality, which may help to understand the mechanisms underlying the gender differences. Nevertheless, it is very important for scientists and clinicians to consider sex differences in sleep research for targeted prevention and intervention to prompt sleep health^35^.

With regard to racial disparity in sleep health, most previous studies focused on White Americans and Black Americans, but few studies in Hispanics, Asians and others^36,37^. In this study of a nationally representative US sample, 64.4% are NH Whites, 11.5% are NH Blacks, 10.1% are Mexican Americans, and 14% of others. Similar to previous findings, NH Blacks exhibited shorter sleep duration, later sleep onset and less sleep efficiency across the lifespan compared to the NH Whites within the same age groups. Moreover, for the first time, we reported sleep characteristics in a population that can nationally represent the Mexican Americans who live across the US. We found that Mexican Americans had the earliest sleep onset, longest sleep duration, but second lowest sleep efficiency (only higher than NH Blacks) compared to other racial groups. Our findings and others suggest that future research on racial disparity in sleep need to take social and cultural backgrounds into account, in addition to biological and genetic differences^36,38^.

There are some limitations to this study. First, although accelerometer recording provides a more objective profile of sleep parameters, the methodology cannot distinguish awake and not moving from sleep^39^. Furthermore, NHANES only released summary counts per minute rather than the raw data. The summary count, MIMS-unit, is designed to reduce the variations between different devices of accelerometer. There is a possibility that the reduced variability from summary counts may overestimate sleep efficiency as well as sleep duration (a product of SPT and sleep efficiency). Sleep efficiency may also have been inflated by our use of the “SPT window” as the denominator for calculating sleep efficiency, as the more traditional denominator of “Time in Bed” was not available. However, this potential overestimation should not have impact on the major finding of this study, which is targeting on the changes of sleep parameters over life span by sex and race. This is reassured by our findings which are in agreement with previous observations of the age, sex and race distribution of sleep duration and sleep quantity collected using self-report questionnaires. Second, sleep diary was not collected during accelerometer recording, therefore, certain parameters of sleep quality such as sleep onset latency cannot be estimated. Third, shift work status/work schedule was not obtained from participants of NHANES 2011–2014 cycle, therefore, a sensitive test by excluding the shift workers cannot be performed.

In conclusion, this study, for the first time, provides changes in objectively measured sleep quality, quantity, and timing over lifespan by sex and race in a US nationally representative sample. Our findings provide valuable insights on the characteristics of sleep habits of residents of the United States and will help guide hygiene research agenda for sleep, including a focus on the social determinants of why different races are differentially impacted by sleep problems.

## Data Availability

The data are publicly available from NHANES website.
